# Forensic Identification: Dental Scan Data Sets of the Palatal Fold Pairs as an Individual Feature in a Longitudinal Cohort Study

**DOI:** 10.3390/ijerph20032691

**Published:** 2023-02-02

**Authors:** Monika Bjelopavlovic, Desiree Degering, Karl Martin Lehmann, Daniel G. E. Thiem, Jochen Hardt, Katja Petrowski

**Affiliations:** 1 Department of Prosthetic Dentistry, University Medical Center of the Johannes Gutenberg, University Mainz, Augustusplatz 2, 55131 Mainz, Germany; 2 Department of Maxillofacial Surgery, University Medical Center of the Johannes Gutenberg, University Mainz, Augustusplatz 2, 55131 Mainz, Germany; 3 Department of Medical Psychology and Medical Sociology, University Medical Center of the Johannes Gutenberg, University Mainz, Duesbergweg 6, 55131 Mainz, Germany

**Keywords:** forensic dentistry, digital technology, palate, patient identification systems, intraoral scanner

## Abstract

The INTERPOL standard for the identification of unknown individuals includes the established primary characteristics of fingerprint, DNA, and teeth. Exposure to noxious agents such as fire and water often severely limits the availability of usable material such as fingerprints. In addition to teeth, the protected oral cavity also houses palatal fold pairs, which are the subject of this study to demonstrate individuality and consequently support identification. **Material and Methods:** In this cohort study, 105 participants’ palates were scanned twice with a dental intraoral scanner (Omnicam SIRONA®) over a 3 month period and were then analyzed using a matching program. The intraindividual and interindividual differences were determined, and the mean values and standard deviations were calculated and presented. **Results:** The intraindividual differences are highly significantly lower than the interindividual differences (*p* < 0.0001). **Conclusions:** Within the limitations of this study, the results suggest that palatal rugae pairs can be considered a highly individual feature and could be considered an identification feature in a young and healthy population.

## 1. Introduction

Global standardization in the identification of unidentified bodies is based on the three primary identifiers: DNA, fingerprints, and teeth. These highly individual characteristics are considered sufficient on their own to pronounce a positive identification. Secondary characteristics, such as physical features in the form of tattoos, carried objects, or personal clothing, do not contribute to the pronouncement of certain identification and must be used in conjunction with a primary identifier [1,2,3,4]. Any doubt must be avoided for the bereaved in order to facilitate the stressful situation through a safe and fast identification process with regard to the mourning process [5,6]. In these situations, experts often face multiple challenges. The acquisition of usable material from the corpse for the identification process, such as fingerprints or DNA, as well as the inspection of remaining teeth, are in the foreground. In particular, the impact of noxious agents such as water, as exemplified by the Ahr valley disaster in 2021 and the experience gained in the 2004 tsunami, show the difficulties in obtaining fingerprints after a long period of immersion [7]. Likewise, the extraction of usable DNA can sometimes be limited, especially in harsh environmental conditions and a long time after death [8]. Thereby, the yield of DNA in post-mortem samples as well as the presence of inhibitors in DNA preparation are potentially limiting factors, as described in the literature [9,10]. Identification via dental status is often crucial and can be performed in a fast and standardized manner if an ante-mortem comparison is available [11,12]. In this context, the oral cavity exhibits a high resistance to any kind of impact and is often a reliable source for the identification process due to the enclosed and well-moisturized space [13,14]. In this sense, even a few remaining teeth can have high information content and can represent individuality with the help of features such as a certain number and position of teeth and the presence or absence of a restoration. This was shown in a study from Spain with 3166 individuals, which found a 0.05% probability that two individuals have an identical combination with only four features (missing, restored, unrestored, and crown) and only 16 teeth present (fully dentate human dentition: 32 teeth) [15]. In addition to the proven individuality of natural teeth, the presence of implants as medical devices adds further characteristic features [16,17]. The demand for implantology is an increasing form of therapy and, in addition to the insertion of characteristic medical devices, also includes radiographs as part of surgical planning as well as surgical and prosthetic follow-up [18]. These patient data could be used to help with identity clarifications. The absence of teeth or other medical devices in the intraoral cavity led to a more difficult identification process in the dental approach. Today, modern dentistry includes digital processes in dental technology such as the production of CAD/CAM-manufactured dentures as well as a digital approach at the dental chair [19,20]. According to the literature, impression-free treatment is favored by many patients, and consequently, there is a consistent trend toward digital treatment in the dental office, which captures intraoral structures using scanning techniques [21]. Consequently, the “digital impression” includes the palatal fold pairs located in the anterior third of the palate. These data would be available in modern practices for important identification measures in the same way as X-ray images or general dental findings, starting the patient’s scanning history from a very young age in orthodontic practices (i.e., for braces and aligner therapy). Furthermore, the acquisition of such data would also be possible from analog plaster models, and consequently, a scanner would not be a prerequisite for a positive application of identification by means of palatal fold pairs [22]. In this context, especially in patients with orthodontic treatment, a possible forensic identification via the palatal fold pairs should be taken with caution since the alteration of these anatomical structures has been described in the literature and, in particular, the length is often described as unstable [23,24]. In the study by Ali et al., it was described that the shape of the palatal fold pairs appears stable after orthodontic treatment, and Weith et al. could demonstrate that the third rugae are the most stable ones. In contrast to these results, the study by Coil et al. recorded the greatest anatomical variance in the third palatal fold pair, and furthermore, it was stated that the use of a constant matching point is doubtful for pre- and post-orthodontic therapy evaluation [25]. However, in cases of traffic accidents or mass disasters, a person’s identity can be difficult to establish, as sometimes only parts of the upper or lower jaw are found and teeth may be lost during the event or post-mortem transportation. Therefore, palatal fold pairs as a well-protected intraoral structure have been investigated in the literature [26]. Dental identification, in particular, is severely lacking in situations and in everyday forensic clinics where the deceased are edentulous. The 5th German Oral Health Study of 2017 shows a halving in edentulism, which nevertheless continues to affect 12.4% of the younger senior group (65–74 years) [27]. Consequently, this group could not be identified based on teeth. Furthermore, depending on the finding situation, removable dentures are not present and cannot often be used for further approaches in the identification chain. The use of palatal fold pairs as an identification marker in these cases is discussed critically in the literature because investigations show that there is a possible change in the structures when removable prostheses are worn [28,29]. Furthermore, cleft palate surgery leads to limitations in the persistence of the palatal rugae as a forensic marker and is investigated in the literature, as is the impact of forced eruption of impacted canines [30,31]. On the contrary, a study by English et al. investigated pre- and post-orthodontic casts of the same individuals. They had to be assigned by different examiners while looking at the palatal fold pairs. As a result, a high accuracy in identifying the correct individual through palatal fold pairs (88–100%) could be demonstrated, although patients underwent orthodontic treatment [32]. These promising approaches stand in contrast to some concerns when including a patient population that underwent a more invasive therapy. Identification by palatal fold pairs alone was judged critically in a review by Jain et al. in orthodontic patients and surgical procedures but was considered a promising forensic marker, especially in burned victims and when teeth are missing [26]. The loss of residual teeth at advanced stages of decay is a common situation. In everyday clinical practice, for example, the absence of teeth also becomes apparent in railroad accidents, although the deceased may be young. In addition, the acquisition of antemortem data is often a difficulty in the identification process and depends on the medical care provided at the scene of the injury and the quality of the documentation [33,34]. Modern, digital dentistry, including intraoral scanning procedures, could contribute to a faster and more efficient antemortem availability and was therefore used in our study design with a fully digital approach. The aim of the present paper is to present an additional reliable measure to extend the diagnostic possibilities in the oral cavity: a highly individual characteristic that is expected to remain constant throughout life and can be demonstrably assigned to a person is to broaden the spectrum of primary identity markers.

The following hypotheses are made:

1. The palatal fold pairs are highly individual and differ significantly from individual to individual;
2. Palatal fold pairs do not differ at different lifetime points.

## 2. Materials and Methods

**Figure 1:**
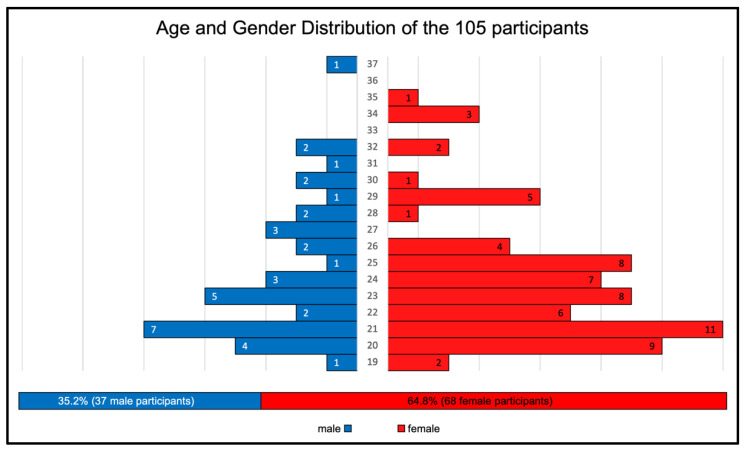
Age distribution of the participants.

The palatal rugae pairs of 105 participants aged between 25 and 38 years were recorded using an intraoral scanner (SIRONA, Omnicam®). The participants were 68 women and 37 men with a mean age of 24 years (minimum 19 years, maximum 37 years; Figure 1; Appendix A).

**Figure 2:**
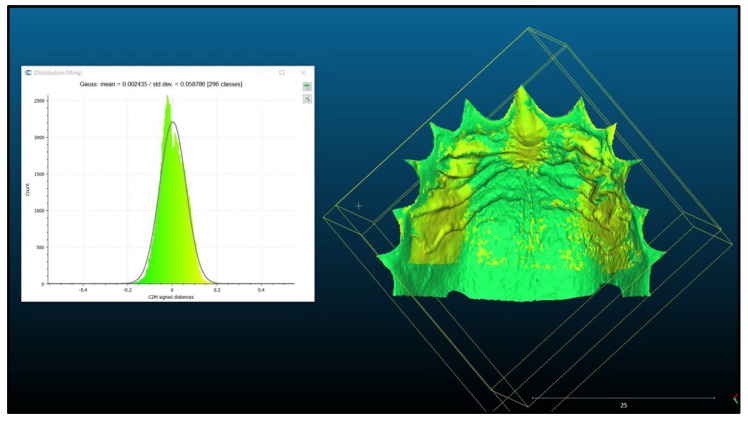
Intraindividual matching.

**Figure 3:**
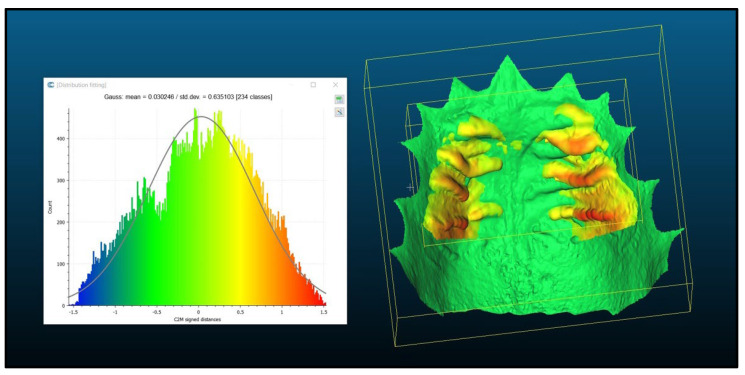
Interindividual matching.

All participants were scanned once and then a second time, exactly three months later. Each participant was assigned a unique ID for pseudonymization. The scan involved the area of the maxillary palate up to the transition from hard to soft palate at the level of the 1st molar. The teeth were only rudimentarily scanned and were not the subject of this examination. For further data processing, the scans were exported as STL files and fed into the open-source software CloudCompare (v. 2 12.0). This is where the matching of the scans took place. Teeth at the gingival margin were manually cut out to prevent distortion of the results. After each cutting, residues were checked, as these are 3D models. Each scan was cut and saved separately. First, each test subject’s scans from different points in time were individually compared. This involved opening the two scans in CloudCompare and performing a rough overlap procedure using the program’s own tool. For this purpose, 4 corresponding points were selected on the scans. These points were placed at prominent locations on the first and second palatal fold pairs, always aiming for the same plane. Based on these points, the program was able to perform a rough overlap procedure automatically. Now the two scans were roughly overlapping, and a fully automated fine alignment could be performed (Figure 2). Following the fine alignment, the distance between the two scans was calculated and output as the mean deviation and standard deviation (in mm). The same procedure was followed for the interindividual comparisons. Pseudo-randomly selected pairs were compared, resulting in a total of 105 interindividual comparisons (Figure 3). An independent statistician who did not know the data randomly assigned pairs with case numbers from 1 to 105 without laying the cases back, i.e., each number occurred exactly once.

The process of pseudo-random selection. First, a pseudo-random number between 1 and 105 was created without laying any cases back, i.e., each number occurred exactly once. They were randomly assigned to the case number and constituted a potential individual for the pairwise comparison. If a certain pair was matched twice, i.e., 15–37 and 37–15, a new run was performed until this did not happen. A total of about 15 runs were needed to gain a first solution where no pair occurred twice. No further run was performed, and these data were then analyzed as displayed in the text. No attempt to obtain significant results was made. There were two rationales behind the procedure chosen. First, testing all possible pairs would have resulted in 5460 comparisons ((k/2) * (k − 1)). Since comparisons were performed manually, this was simply not possible. Second, if two groups with 5460 and 105 cases were compared, the first group would have a much stronger influence on the statistical test. Therefore, we formed the group for the interindividual test in a similar sample size as the group for the intraindividual comparison The complete procedure in the evaluation was performed twice by the investigator with all intra- and interindividual pairs of participants. The length and shape of the palatal fold pairs were not investigated in this study. Subsequently, an evaluation took place with the help of the program STATA 17 (STATACORP 2022, Revision 10) using a *t*-test.

*Inclusion criteria:*

Healthy participants who consented to be scanned.

*Exclusion criteria:*

General history that includes anomalies of the palate (cleft lip and palate; tumors; and syndromes).

## 3. Results

**Figure 4:**
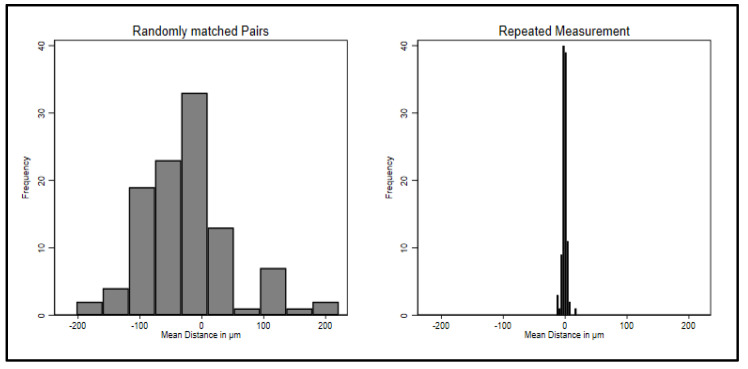
Mean distances for inter- and intraindividual comparisons.

The analysis program returns two values: the mean distances (sum of distances/n) of the two scans of the palate and the standard deviation [squared root (sum ($x_{i}$ − $\bar{x}$)²/n] of the distances of the two scans. Histograms of the mean distances and standard deviations are displayed for the two groups in Figure 4 (the output was converted from mm to μm). The means of both groups are close to zero, but repeated scans (from one participant) show much less variance than randomly assigned pairs (from two different participants). This is exactly what was expected, because in the repeated measurement case there is only random measurement error, whereas in the randomly matched pairs there is additional random measurement error and a large amount of variance from matching. Since they are distances on a three-dimensional sheet measured in μm, both should be normally distributed around a mean of zero.

**Figure 5:**
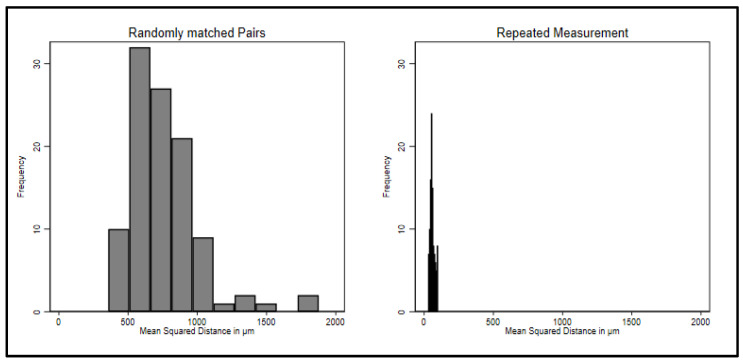
Mean squared distances for inter- and intraindividual comparisons.

Figure 4 and Figure 5 show the squared mean distances and their standard deviations. A *t*-test informs that the mean squared distances in repeated measurement are highly significantly smaller than in matched pairs (*t* = 25.88, df = 209, and the *p* value has 71 zeros after the decimal). Cohen’s d for the squared distances is d = 3.55 and represents a very large effect in Cohen’s classification [35]. As a cut-off between repeated measurements of the same person and interindividual differences, we preliminarily suggest 300 μm. No case of the randomly matched pairs has a squared difference smaller than 300 μm, and no case of the repeated measurement has a value larger than 300 μm. This may be revisited in future research.

## 4. Discussion

In this study, it could be proven that the palatal fold pairs are highly individual and related to the measurement period constant in their expression in the life of a person. Concluding from the results, these are to be classified as individual characteristics, that differ highly significantly and individually. This has even been demonstrated in the literature while investigating the palatal fold pairs of siblings. A study by Chong et al. showed the expression of genetic similarity between siblings in the palatal fold pairs, which nevertheless showed individuality and differed interindividually [35]. Even in twins, significant palatal fold individuality could be demonstrated. Twigs twins, as well as monozygotic twins, showed differences and could be clearly distinguished from each other based on the palatal fold pairs. In 2020, Simon et al. also followed up on this patient group with scan data and were able to demonstrate the special role of palatal fold pairs regarding forensic identification [36]. A study by Saraf et al. showed the possibility of sex determination by palatal fold pairs and their gender-specific expression regarding height, forms, and size [37]. Furthermore, a study performed by Bailey et al. in 1996 using plaster models investigated the structure of the palatal fold pairs during extraction procedures. Models were made before and after extraction in the premolar region of adult patients between 18 and 36 years of age in order to determine possible changes in the rugae that may have occurred as a result of the surgical procedure [38]. Looking at surgical procedures as a possible influence on the anatomic structure of the palatal fold pairs, it was shown that an extraction of teeth showed no significant impact on the rigid and elevated design and dimensions of the pairs. The results of this study were supported by a review by Jain et al. (2012), which also emphasized the individuality of the rugae palatinae but raised concerns about permanence at different time points [26]. The persistence of palatal anatomic structures was demonstrated by intraindividual comparisons at different times in our study, but the three-month observation period must be viewed critically. In contrast, the longitudinal study by Christou et al., which investigated the change in the palatal fold pairs after transversal orthodontic treatment, was conducted over a period of 4 years [39]. The 23 patients consisted of 10 adults and 13 adolescents. Both groups showed vertical changes in the first, second, and third palatal fold pairs after orthodontic therapy but did not differ significantly, although the adolescents showed greater changes over time than adults. However, it must be considered that the sample size of the study was relatively small with n = 23. Another study by Kapoor et al. could demonstrate the statistically significant changes in the palatal fold pairs after mid-palatal expansion treatment, which included transversal and medial aspects, especially in the third palatal fold pair [40]. Changes after orthodontic treatment concerning expansion were also described by Saadeh et al. [41], and the detected changes should be critically considered in terms of forensic identification possibilities, which were also investigated in a study by Deepak et al. [42]. Future studies should evaluate possible palatal anatomical expressions that would be of permanent stability, despite orthodontic expansion. However, after the completion of orthodontic therapy, a final scan is performed, which would preserve the change in structures and could be provided as a data set in terms of identification without having to resort to the falsified initial situation. The rugae of the patient collective in the mentioned studies were all evaluated manually and, in contrast to our study, were measured two-dimensionally at the most mesial and distal points of the pairs, using marker points on the plaster models. Another study also used the conventional casting technique and afterwards scanned and matched the plaster models digitally [43]. This method was also applied in a study by Makrygiannakis et al. in 2022 with 20 patients while looking at the three-dimensional structure of the palatal fold pairs and showing their complexity in forensic cases [44]. In contrast, in the present study, we were able to work on a three-dimensional model analysis in the computer program after direct digital recording of the intraoral situation. This further development in the current dental routine would lead to considerable time savings in the context of identification measures. The fact that modern digital dentistry includes intraoral scans should also be considered in terms of data availability when it comes to identification processes. Antemortem data acquisition is a crucial and time-consuming factor in terms of identification, and the quality of patient-related data material or casts can differ from dentist to dentist [33]. Intraoral scan data sets like those investigated in our study could provide standardized and high-quality data that does not have material-related inaccuracies like in gips cast models.

*Limitations:* Currently, the method described here has the following limitations. Preparation of the scans must be done manually. This is (1) time-consuming, (2) always error-prone, and (3) does not allow fully automated comparisons via computer. Indeed, the person preparing the scans (4) needs to have some experience to obtain optimal results. The patient population contains a collective of young and healthy adults, not representing a population (5), and shows the invariability of the palatal fold pairs based on a life section of 3 months (6). Consequently, our results should be tested on a larger patient population, including elderly participants as well as patients with a broad general history and anomalies in the palate. The population should be observed and scanned during a longer life period. Furthermore, the applicability to deceased individuals should be the subject of future investigation in order to provide another primary identification feature, for instance, in case of a mass disaster. Analogous to the human fingerprint, the palatal fold pairs could also contribute to a secure identification while using the scan data that is acquired during modern dental treatment. Progressive developments in digitalization, particularly in dentistry, would probably support this process. Another possible advancement would be the creation of a fully automated comparing procedure, for example, by utilizing artificial intelligence. However, it must be admitted that such a procedure is currently not available, and its development will probably not become possible without incurring costs.

## 5. Conclusions

Dental identification depends on the presence of teeth and a sufficient number of characteristic findings. As a promising candidate, the palatal fold pairs were investigated regarding individuality and stability while using a dental intraoral scanner, analogous to modern dental clinical practice.

Within the limitations of this study, the following conclusions are drawn:

1. Palatal fold pairs are highly individual and differ significantly from individual to individual. This hypothesis can be confirmed;
2. Palatal fold pairs do not differ at different lifetime points. This hypothesis cannot be confirmed conclusively. In our study, it can only be confirmed for a lifetime of 3 months;
3. The results of our study allow the assumption that the palatal fold pairs could be suitable as a primary identification feature analogous to a fingerprint in a young and healthy population.

## Figures and Tables

**Figure A1:**
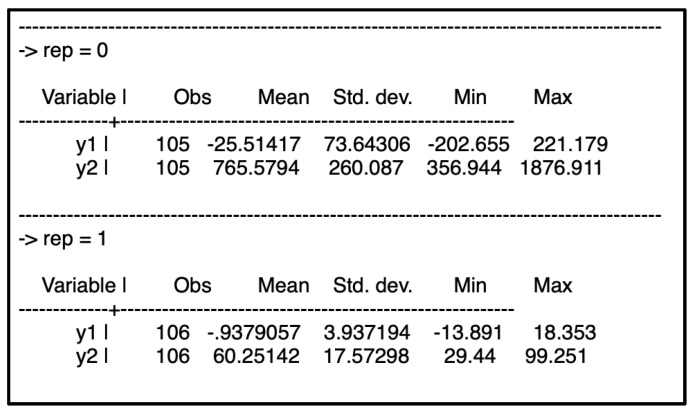
Descriptive statistics for inter- and intraindividual comparisons.

## Data Availability

The data from this study were part of the dissertation paper from D.D. Data can be seen in Figure 1, Figure 2, Figure 3 and Figure 4. The scan data is available upon request.
